# Supplementary material for: Hotspots and super-spreaders: Modelling fine-scale malaria parasite transmission using mosquito flight behaviour
Source: PLoS Pathog. 2022 Jul 6;18(7):e1010622. doi: 10.1371/journal.ppat.1010622 (PMC9292116; doi:10.1371/journal.ppat.1010622)
Supplement: S1 File — (PDF) [file ppat.1010622.s001.pdf]

S1 File

Full explanation of the mosquito flight parameters

Upwind, random and downwind mosquito flight movements have been described for African mosquitoes since 1960s. In his field experiments, Snow provided the first evidence that upwind and downwind flights are affected by flight height, wind speed and direction [1-3].

Number of days prior to infection (DPI) was set between day 21 and day 1 prior to the house survey. This time accounts for the full intrinsic incubation period, uncompleted extrinsic incubation period (if not completed during the days of flight) and uncertainty in RDT test. We are only considering infections between houses and not within the house. The DPI, in combination with the number of days of flight (see below), allows for the completion of both the intrinsic [4] and extrinsic [5] incubation periods.

Number of days of flight (DoF) was set between 1 to 18 days, ending from the number of days prior to the infection date (e.g. if infection occurred on the 29th of July and the number of days prior to infection was 10, then the mosquito arrived in the house on the 19th of July with flight starting from the departing house any day from the 2nd of July; which means that between the 19th of July and the 29th of July the infectious bite and incubation period were completed). The sum of maximum number of days previous to infection and maximum days of flight (DPI+DoF = 39 days) is within the lifespan of *Anopheles* mosquitoes [6]. MALSWOTS does not model the lifespan of the mosquito and assumes homogenous flight capabilities within the DoF + DPI period. S1 Fig shows how the parameters DPI and DoF operate within MALSWOTS.

Numbers of hours of continuous flight is set at 1h per day (or time of the day period). Tethered flight experiments have shown that *An. gambiae* and *An. atroparvus* can continuously fly for 1 to 4 hours, although most flights lasted less than 1 hour [7].

The *maximum distance of flight in one day* is 1000 m (therefore any distance between 0 and 1000 m is accepted as day flight). This value reflects the dispersal capacity of *An. gambiae, An. arabiensis* and *An. funestus*. Mark-release-recapture studies estimate an average distance between 150m and 500m away from the release location, although many mosquitoes are still found well over 1000m one day post-release [8-13]. Here, we do not consider the long-range migratory behaviour identified in Sahelian mosquitoes [14] as we are considering climate and environment at near soil conditions and our primary interest is movement between houses. Long-range dispersion above 1 km in our setting is thus more likely to happen in steps over multiple days instead of single flights.

A large body of literature is available on mosquito's attractants (primarily carbon dioxide) and their capacity to stimulate upwind mosquito movement [15-21]. However, limited information is available for the *distance at which the mosquito can detect the host*. Here, we set this distance at 50m according to previous work [22,23].

Distance around each house (essentially a buffer zone around the house that determines a successful connection) permits outdoor malaria transmission [24] and flexibility in the model. This parameter was fixed at 15 m, lower than those proposed elsewhere [25] to allow for houses separated by as little as 30 m to have a different infection status.

Anopheles *flight speed* is heterogeneously reported and is generally around 0.3 m/s but can be as high as 1m/s for short periods [26-28]. However, these values do not discriminate between mosquito flight and wind propulsion. Therefore, we have set this parameter to the average value of 0.1 m/s. In upwind movement this value of 0.1 m/s is reduced according to the wind speed (e.g. if the wind speed is 0.05 m/s, the upwind flight speed is 0.05 m/s) while in downwind movement the mosquito flight speed adds to the wind speed (e.g. if the wind speed is 0.1 m/s, the downwind flight speed is 0.2 m/s).

High winds can inhibit flight and low numbers of mosquitoes are caught at winds above 40 km per hour [3]. We therefore set the *wind limits* for all flight at 11 m/s based on limits for

upwind flight [2], although the average hourly wind speed (and gust speed) rarely reached this value within the MMP area.

Finally, wind direction is highly variable [29] and a *tolerance around the average direction* is often applied in mosquito flight modelling [30]. For the present analysis we set this tolerance to 45 degrees around the hourly average. This value is consistent with the capacity of the mosquitoes to adjust their flight if odour plumes come from a different direction [30].

MALSWOTS algorithm

As in SWOTS (see [31] for a detailed description of the calculations), MALSWOTS data preparation starts by transforming the wind plumes into mosquito trajectories for the different mosquitoes' movements (by using the conditions stated in mosquito flight parameters section): downwind, upwind, random, downwind and random, and upwind and random; while the potential infections of houses are transformed into infection connections (see MALSWOTS algorithm flow below). Various controls are performed at the beginning and if necessary infected locations that are too close to uninfected locations are removed (i.e. if the infected and uninfected house are within 65 m - the sum of the distance at which the mosquito can detect the host and the distance around each house).

Taking each infected house in turn, from the date of its infection, the connections were created for every other house infected at some future date (up to a maximum of DPI+DoF days). A maximum distance limitation was also applied (see mosquitoes flight parameters). The infection connections form a matrix consisting of rows representing houses and the five following columns: average number of days to potentially infect other houses; mean distance of connections along the longitude; mean distance of connections along the latitude; average connection speed in meters per days; and frequency of connections along the average connection directions. These five columns were converted from a five dimension Euclidian space to a spherical system (which allow the standardisation of the dimensions) [32]. The same matrix is created for each mosquito trajectory. Mosquito trajectories are the sum of geometric vectors, starting from the infected house in the first day and from the endpoint of the geometric vector the subsequent day (the head-to-tail sum rule). Therefore, mosquito trajectories do not necessarily end in an infected or uninfected house; they can end in any point of the geographic area. Their length and direction depend on wind speed and direction as described above. Mosquitoes are assumed to use a variety of movement strategies, up- and down-wind, random and mixed movements, the latter allowing for a random component in their daily flight. Assuming each of these strategies in turn, and a mixture of these strategies, five resulting mosquito trajectories matrices were produced: downwind, upwind, random, mixed downwind and random, and mixed upwind and random. Between each mosquito movement matrix and infection connections matrix, both in spherical space, the vectorial correlation is calculated following the approach proposed by [33] and fully described in [31].

The process described above was repeated 450 times, each time with a different combination of DoF (up to 18 days) and DPI (up to 21 days). In addition, it was repeated for different time periods: 6.00pm-10.59pm, 11.00pm-3.59am, 4.00am-8.59am, and finally for the entire period studied 6.00pm-8.59am where mosquitoes are assumed flying for the full length of the time period; therefore providing 9000 correlations between infection connections and mosquitoes movements (450 combinations x 4 day periods x 5 mosquitoes movements). The day periods were chosen as representative of the Malawian *Anopheles* biting pattern [34]. Each calculated correlation was tested via spatial bootstrapping to ensure sensitivity and to reduce error [35]. The spatial bootstrapping allows for the definition of the level of significance, here taken to be 99%.

The (significant) correlations were weighted [36] by the number of surviving mosquitoes (see below for the details of the survival model). The subsequent analysis is applied with the parameters associated to the top correlations (or the theoretical best if preferred). The analysis

is re-run to describe mosquito trajectories from house to house and included uncertainty in wind speeds and directions. For the latter, a stochastic component for both wind speed and direction were added. In each simulation, a new value of wind speed and direction for each grid node is sampled. The wind speeds are assumed to be multivariate log-normal distributed, where the mean is a vector containing each grid node's average wind speed for the considered time period (day periods defined above) while the variance is fixed to 1. Assuming a multivariate distribution allows the inclusion of correlations between wind speeds at different grid nodes [37]. The wind directions are assumed to be normally distributed, with the mean at each grid nodes equal to the average wind direction for the considered time period and the variance equal to 1. This normal distribution is wrapped in order to have values between $-\pi$ and π [38]. It is important to note that each focal area has been converted into grids with each cell containing the values of wind and other environmental variables for that day or time of the day (see below).

At each simulation, from a new (sampled) set of wind speeds and directions, the five types of mosquito trajectories were re-calculated. The total number of simulations was 1,000. For each newly infected house we therefore had a set of connections from different, previously infected houses. The MALSWOTS algorithm flow (modified from [31]) is provided below.

MALSWOTS

0. Hourly wind speed and direction were converted to their mean values for the five periods within each day. A survival model is applied to mosquito trap counts using environmental variables (temperature and precipitation). For each cell of the grid the probability to find an infected house is calculated (based on the ratio between number of infected and uninfected houses).

1. For each day period or time of day (TOD):
 1.1. For each number of days previous to infection (DPI):
  1.1.1. For each number of days of flight (DoF):
   1.1.1.1. Rescale the infection date, *t*, to: *t*' = *t* - (DPI+DoF).
   1.1.1.2. Select all the houses separated by a time interval of 1 to DoF days by applying the following condition: 0<differences(*t*) <=DoF
   1.1.1.3. At each house calculate the average disease trajectory (geometric vector) *d*.
   1.1.1.4. For each mosquito movement:
    1.1.1.4.1 At each house calculate the average departing mosquito movement trajectory (geometric vector) *w* using the mosquito flight parameters and wind speed and direction.
    1.1.1.4.2 Convert **D** (containing all the house *d*) and **W** (containing all the house *w*) matrices into spherical systems, **D**' and **W**'.
    1.1.1.4.3 Calculate the weighted correlation between **D**' and **W**' and its significance (via spatial bootstrapping).
   Close 1.1.1.4. (mosquito movement).
  Close 1.1.1. (DoF).
 Close 1.1. (DPI).
Close 1. (TOD)

2. Take all the weighted correlations produced in 1.1.4. for all five movements, DoF, DPI and day periods and rank them from the largest to the smallest. Take the top *n*. Each selected correlation is associated with an optimal value of day period (TOD*), DoF (DoF*) and DPI (DPI*).

3. Carry out stochastic simulation to provide houses connections' probabilities.
 3.1. For each parameterisation of the *n* selected:
  3.1.1. Rescale *t* according to DoF* and DPI* and take the winds associated with TOD*.
  3.1.2. For each simulation:
   3.1.2.1. Sample wind speeds and directions for each grid node and day from prior distributions.
   3.1.2.2. For each type of mosquito movement:
    3.1.2.2.1. For each value in the range 1 to DoF*:
     3.1.2.2.1.1. At each house calculate the average departing mosquito movement trajectory *w* as in 1.1.1.4.1.
     3.1.2.2.1.2. If the trajectory reaches an infected house within a DoF* time from the departing trajectory house, the trajectory is deemed successful and a connection is established.
    Close 3.1.2.2.1. (DoF*).
   Close 3.1.2.2. (type of movement).
  Close 3.1.2. (for each simulation).
 Close 3.1. (from 1 to *n*)

4. Calculate the final probabilities:
 4.1. Daily survival probability for each connection
 4.2. From the survival probability, the probability of presence of an infected house in the cell grid and number of connections, the probabilities of a house been infected from any house, an house been infected by another house, and an house been infected by a type of mosquito movement are calculated.
 4.3. Estimate the type of mosquito movement most likely responsible for carrying the infection from the infecting to the infected house.

5. STOP.

The probability of infection by the different types of mosquito movement at a house and the cumulative probability of a house escaping infection (a cross-product of the probability of each movement infecting the house) are obtained from the survival of the mosquito at certain environmental conditions (i.e. temperature and rainfall) and time of flight. Mosquito survival is calculated by employing an exponential model with the number of mosquitoes caught in each trap Poisson distributed with the mean proportional to time and rate linearly dependent on temperature and precipitation:

$$X \sim Poisson(\mu)$$

$$log\mu = \log(t) + \beta_0 + \beta_1 Prec + \beta_2 Temp$$

where *X* is the number of mosquitoes caught at time *t*; time, *t*, is considered as offset; β_0 is the intercept; β_1 is the coefficient for precipitation; and β_2 is the coefficient for temperature. The survival model was restricted to precipitation and temperature only since relative humidity and soil water content were not found statistically significant predictors when added to the model or in alternative to rainfall and temperature.

These probabilities of survival (and therefore infection) are multiplied by the probability of finding infected houses in the area (grid cell) and by the respective frequency of movements. The latter product is then adjusted by the total number of potential connections (assuming one day is equal to one connection per mosquito movement) and total number of simulations to obtain the final probabilities of (i) a house being infected from all surrounding infectious houses (since one house is likely to be infected by mosquitoes arriving from multiple houses with different movement types), (ii) a house being infected by a specific house, and (iii) a house being infected by a type of mosquito movement (see S2 Fig).

At present, the probability of a house being infected does not account for housing characteristics (number of individuals in the house, house type etc), other than the two climate variables used to model mosquito survival. However, the current framework does allow inclusion of house-dependent factors or additional environmental (including climate) variables if the data is available.

References

1 Snow, W. F. The direction of flight of mosquitoes (Diptera, Culicidae) near the ground in West African savanna in relation to wind direction, in the presence and absence of bait. *Bull. Entomol. Res* **65**, 555-562, doi:10.1017/S0007485300006234 (1976).

2 Snow, W. F. The height and direction of flight of mosquitoes in West African savanna, in relation to wind speed and direction. *Bull. Entomol. Res* **67**, 271-279, doi:10.1017/S0007485300011081 (1977).

3 Snow, W. F. Field estimates of the flight speed of some West African mosquitoes. *Annals of Tropical Medicine & Parasitology* **74**, 239-242, doi:10.1080/00034983.1980.11687334 (1980).

4 Zhang, Y., Liu, S. & Bai, Z. A periodic malaria model with two delays. *Physica A: Statistical Mechanics and its Applications* **541**, doi:10.1016/j.physa.2019.123327 (2020).

5 Stopard, I. J., Churcher, T. S. & Lambert, B. Estimating the extrinsic incubation period of malaria using a mechanistic model of sporogony. *PLoS Comput Biol* **17**, e1008658, doi:10.1371/journal.pcbi.1008658 (2021).

6 Clements, A. N. & Paterson, G. D. The Analysis of Mortality and Survival Rates in Wild Populations of Mosquitos. *J Appl Ecol* **18**, 373-399, doi:Doi 10.2307/2402401 (1981).

7 Kaufmann, C. & Briegel, H. Flight performance of the malaria vectors Anopheles gambiae and Anopheles atroparvus. *J Vector Ecol* **29**, 140-153 (2004).

8 Gillies, M. T. Studies on the dispersion and survival of Anopheles gambiae Giles in East Africa, by means of marking and release experiments. *B Entomol Res* **52**, 99-127 (1961).

9 Charlwood, J. D. Studies on the bionomics of male Anopheles gambiae Giles and male Anopheles funestus Giles from southern Mozambique. *J Vector Ecol* **36**, 382-394, doi:10.1111/j.1948-7134.2011.00179.x (2011).

10 Midega, J. T. *et al.* Estimating dispersal and survival of Anopheles gambiae and Anopheles funestus along the Kenyan coast by using mark-release-recapture methods. *Journal of Medical Entomology* **44**, 923-929, doi:Doi 10.1603/0022-2585(2007)44[923:Edasoa]2.0.Co;2 (2007).

11 Minakawa, N., Seda, P. & Yan, G. Y. Influence of host and larval habitat distribution on the abundance of African malaria vectors in western Kenya. *Am J Trop Med Hyg* **67**, 32-38, doi:DOI 10.4269/ajtmh.2002.67.32 (2002).

12 Thomas, C. J., Cross, D. E. & Bogh, C. Landscape Movements of Anopheles gambiae Malaria Vector Mosquitoes in Rural Gambia. *Plos One* **8**, doi:10.1371/journal.pone.0068679 (2013).

13 Verdonschot, P. F. M. & Besse-Lototskaya, A. A. Flight distance of mosquitoes (Culicidae): A metadata analysis to support the management of barrier zones around rewetted and newly constructed wetlands. *Limnologica* **45**, 69-79, doi:10.1016/j.limno.2013.11.002 (2014).

14 Huestis, D. L. *et al.* Windborne long-distance migration of malaria mosquitoes in the Sahel. *Nature* **574**, 404-+, doi:10.1038/s41586-019-1622-4 (2019).

15 Healy, T. P., Copland, M. J. W., Cork, A., Przyborowska, A. & Halket, J. M. Landing responses of Anopheles gambiae elicited by oxocarboxylic acids. *Med Vet Entomol* **16**, 126-132, doi:DOI 10.1046/j.1365-2915.2002.00353.x (2002).

16 Frei, J., Krober, T., Troccaz, M., Starkenmann, C. & Guerin, P. M. Behavioral response of the malaria mosquito, Anopheles gambiae, to human sweat inoculated with axilla bacteria and to volatiles composing human axillary odor. *Chem Senses* **42**, 121-131, doi:10.1093/chemse/bjw106 (2017).

17 Costantini, C. *et al.* Electroantennogram and behavioural responses of the malaria vector Anopheles gambiae to human-specific sweat components. *Med Vet Entomol* **15**, 259-266, doi:DOI 10.1046/j.0269-283x.2001.00297.x (2001).

18 Hawkes, F., Young, S. & Gibson, G. Modification of spontaneous activity patterns in the malaria vector Anopheles gambiae sensu stricto when presented with host-associated stimuli. *Physiol Entomol* **37**, 233-240, doi:10.1111/j.1365-3032.2012.00838.x (2012).
19 Smallegange, R. C. *et al.* Identification of candidate volatiles that affect the behavioural response of the malaria mosquito Anopheles gambiae sensu stricto to an active kairomone blend: laboratory and semi-field assays. *Physiol Entomol* **37**, 60-71, doi:10.1111/j.1365-3032.2011.00827.x (2012).
20 Dekker, T., Takken, W. & Braks, M. A. H. Innate preference for host-odor blends modulates degree of anthropophagy of Anopheles gambiae sensu lato (Diptera : Culicidae). *Journal of Medical Entomology* **38**, 868-871, doi:Doi 10.1603/0022-2585-38.6.868 (2001).
21 Batista, E. P. A. *et al.* Videographic analysis of flight behaviours of host-seeking Anopheles arabiensis towards BG-Malaria trap. *Plos One* **14**, doi:10.1371/journal.pone.0220563 (2019).
22 Gillies, M. T. & Wilkes, T. J. The range of attraction of single baits for some West African mosquitoes. *Bull Entomol Res* **60**, 225-235, doi:10.1017/S000748530004075X (1970).
23 Lorenz, L. M. *et al.* Taxis assays measure directional movement of mosquitoes to olfactory cues. *Parasite Vector* **6**, doi:10.1186/1756-3305-6-131 (2013).
24 Mburu, M. M. *et al.* Biting patterns of malaria vectors of the lower Shire valley, southern Malawi. *Acta Trop* **197**, 105059, doi:10.1016/j.actatropica.2019.105059 (2019).
25 Sturrock, H. J. *et al.* Reactive case detection for malaria elimination: real-life experience from an ongoing program in Swaziland. *PLoS One* **8**, e63830, doi:10.1371/journal.pone.0063830 (2013).
26 Cohnstaedt, L. W. & Allan, S. A. Effects of sublethal pyrethroid exposure on the host-seeking behavior of female mosquitoes. *J Vector Ecol* **36**, 395-403, doi:10.1111/j.1948-7134.2011.00180.x (2011).
27 Angarita-Jaimes, N. C. *et al.* A novel video-tracking system to quantify the behaviour of nocturnal mosquitoes attacking human hosts in the field. *J R Soc Interface* **13**, doi:10.1098/rsif.2015.0974 (2016).
28 Reynolds, D. R. *et al.* Atmospheric transport of mosquitoes in northeast India. *Med Vet Entomol* **10**, 185-186, doi:DOI 10.1111/j.1365-2915.1996.tb00727.x (1996).
29 Bidlingmayer, W. The measurement of adult mosquito population changes- some considerations. *Journal of the American Mosquito Control Association* **1**, 328-348 (1985).
30 Cummins, B., Cortez, R., Foppa, I. M., Walbeck, J. & Hyman, J. M. A Spatial Model of Mosquito Host-Seeking Behavior. *Plos Computational Biology* **8**, doi:10.1371/journal.pcbi.1002500 (2012).
31 Sedda, L. *et al.* A new algorithm quantifies the roles of wind and midge flight activity in the bluetongue epizootic in northwest Europe. *Proceedings of the Royal Society B-Biological Sciences* **279**, 2354-2362, doi:10.1098/rspb.2011.2555 (2012).
32 Henderson, D. W. & Taimiņa, D. *Experiencing Geometry: In Euclidean, Spherical, and Hyperbolic Spaces*. (Prentice Hall, 2001).
33 Stephens, M. A. Vector Correlation. *Biometrika* **66**, 41-48, doi:DOI 10.1093/biomet/66.1.41 (1979).
34 The PMI Vector Link Project. (Abt Associates Inc, Rockville, MD, USA, 2019).
35 Loh, J. M. & Stein, M. L. Spatial bootstrap with increasing observations in a fixed domain. *Stat Sinica* **18**, 667-688 (2008).
36 Bland, J. M. & Altman, D. G. Calculating correlation coefficients with repeated observations: Part 2--Correlation between subjects. *BMJ* **310**, 633, doi:10.1136/bmj.310.6980.633 (1995).
37 Aitchison, J. *The Statistical Analysis of Compositional Data*. (Blackburn Press, 2003).
38 Nadarajah, S. & Zhang, Y. Wrapped: An R package for circular data. *PLoS One* **12**, e0188512, doi:10.1371/journal.pone.0188512 (2017).
