# Supplementary material for: Hotspots and super-spreaders: Modelling fine-scale malaria parasite transmission using mosquito flight behaviour
Source: PLoS Pathog. 2022 Jul 6;18(7):e1010622. doi: 10.1371/journal.ppat.1010622 (PMC9292116; doi:10.1371/journal.ppat.1010622)
Supplement: S1 Table — Data representation on the form m(q,s) where ‘m’ is mean number of mosquitoes collected per night in one trap x 1000, ‘q’ is the standard deviation x 1000, and ‘s’ is the sum x 1000. s.l. refers to specimens that did not amplify in PCR, therefore species identification is based only on morphology. (-) and (+) indicate results of the qPCR for P. falciparum sporozoites. (DOCX) [file ppat.1010622.s002.docx]

S1 Table. Entomological collection summaries for each focal area. Data representation on the form m(q,s) where ‘m’ is mean number of mosquitoes collected per night in one trap x 1000, ‘q’ is the standard deviation x 1000, and ‘s’ is the sum x 1000. s.l. refers to specimens that did not amplify in PCR, therefore species identification is based only on morphology. (-) and (+) indicate results of the qPCR for *P. falciparum* sporozoites

| Species | Focal area A | Focal area B | Focal area C |
| --- | --- | --- | --- |
| *An. gambiae* s.s. male | 0 | 0 | 0 |
| *An. gambiae* s.s. female (-) | 0 | 2.111 (46, 3000) | 0.727 (27, 1000) |
| *An. gambiae* s.s. female (+) | 0 | 0 | 0.727 (27, 1000) |
| *An. arabiensis* male | 0 | 0.704 (26, 1000) | 1.454 (38, 2000) |
| *An. arabiensis* female (-) | 0.652 (25,1000) | 54.891 (309, 76000) | 22.545 (296, 31000) |
| *An. arabiensis* female (+) | 0 | 2.815 (65, 3000) | 2.910 (85, 4000) |
| *An. gambiae* s.l. male | 0 | 0 | 0 |
| *An. gambiae* s.l. female (-) | 0 | 7.741(95, 10000) | 0.727 (27, 1000) |
| *An. gambiae* s.l. female (+) | 0 | 0 | 0 |
| *An. funestus* s.s. male | 0 | 0 | 0 |
| *An. funestus* s.s. female (-) | 0 | 27.445 (249, 39000) | 42.181 (383, 58000) |
| *An. funestus* s.s. female (+) | 0 | 2.111 (46, 1000) | 1.454 (38, 2000) |
| *An. funestus* s.l. male | 0 | 0 | 0 |
| *An. funestus* s.l. female (-) | 0 | 0.703 (26, 1000) | 2.181 (60, 3000) |
| *An. funestus* s.l. female (+) | 0 | 0 | 0 |
|  |  |  |  |
| Total *Anopheles* captured | 1 | 134 | 103 |
| Number of trap collections | 1520 | 1407 | 1375 |
