# Supplementary material for: Hotspots and super-spreaders: Modelling fine-scale malaria parasite transmission using mosquito flight behaviour
Source: PLoS Pathog. 2022 Jul 6;18(7):e1010622. doi: 10.1371/journal.ppat.1010622 (PMC9292116; doi:10.1371/journal.ppat.1010622)
Supplement: S2 Table — ^ confidence intervals. All parameters are calculated over the whole study period. (DOCX) [file ppat.1010622.s003.docx]

S2 Table. MALSWOTS (without mosquito survival probabilities) estimated optimal parameters and transmission summaries obtained from the top 5% of the models ranked by largest correlation. ^ confidence intervals. All parameters are calculated over the whole study period.

| Parameters | Focal area A | Focal area B | Focal area C |
| --- | --- | --- | --- |
| Days previous of infection (DPI) | 9 (1, 21)^ | 7 (1, 19)^ | 7 (1, 21)^ |
| Days of mosquito flight (DoF) | 16 (8, 16)^ | 9 (9, 15)^ | 14 (13, 16)^ |
| Days to new infection | 23 (14, 36)^ | 18 (10, 33)^ | 21 (15, 36)^ |
| Selected parameters 6pm-10pm (%) | 58.54 | 40.42 | 68.75 |
| Selected parameters 11pm-3am (%) | 14.63 | 23.40 | 22.92 |
| Selected parameters 4am-8am (%) | 26.83 | 36.17 | 8.33 |
| Selected parameters 6pm-8am (%) | 34.92 | 38.15 | 33.33 |
