# Supplementary material for: Hotspots and super-spreaders: Modelling fine-scale malaria parasite transmission using mosquito flight behaviour
Source: PLoS Pathog. 2022 Jul 6;18(7):e1010622. doi: 10.1371/journal.ppat.1010622 (PMC9292116; doi:10.1371/journal.ppat.1010622)
Supplement: S3 Table — (DOCX) [file ppat.1010622.s004.docx]

S3 Table. Cross validation results for infected houses using MALSWOTS without mosquito survival probabilities.

| Focal area | % correct prediction | Probability before | Probability around time of infection* | Probability after |
| --- | --- | --- | --- | --- |
| A | 55 | 0.30 | 0.49 | 0.21 |
| B | 77 | 0.11 | 0.74 | 0.15 |
| C | 50 | 0.27 | 0.41 | 0.32 |

* The probability around time of infection is defined as the probability to be infected during one DPI+DoF before and after a RDT positive test.
