# Supplementary figures and images for: Hotspots and super-spreaders: Modelling fine-scale malaria parasite transmission using mosquito flight behaviour
Source: PLoS Pathog. 2022 Jul 6;18(7):e1010622. doi: 10.1371/journal.ppat.1010622 (PMC9292116; doi:10.1371/journal.ppat.1010622)

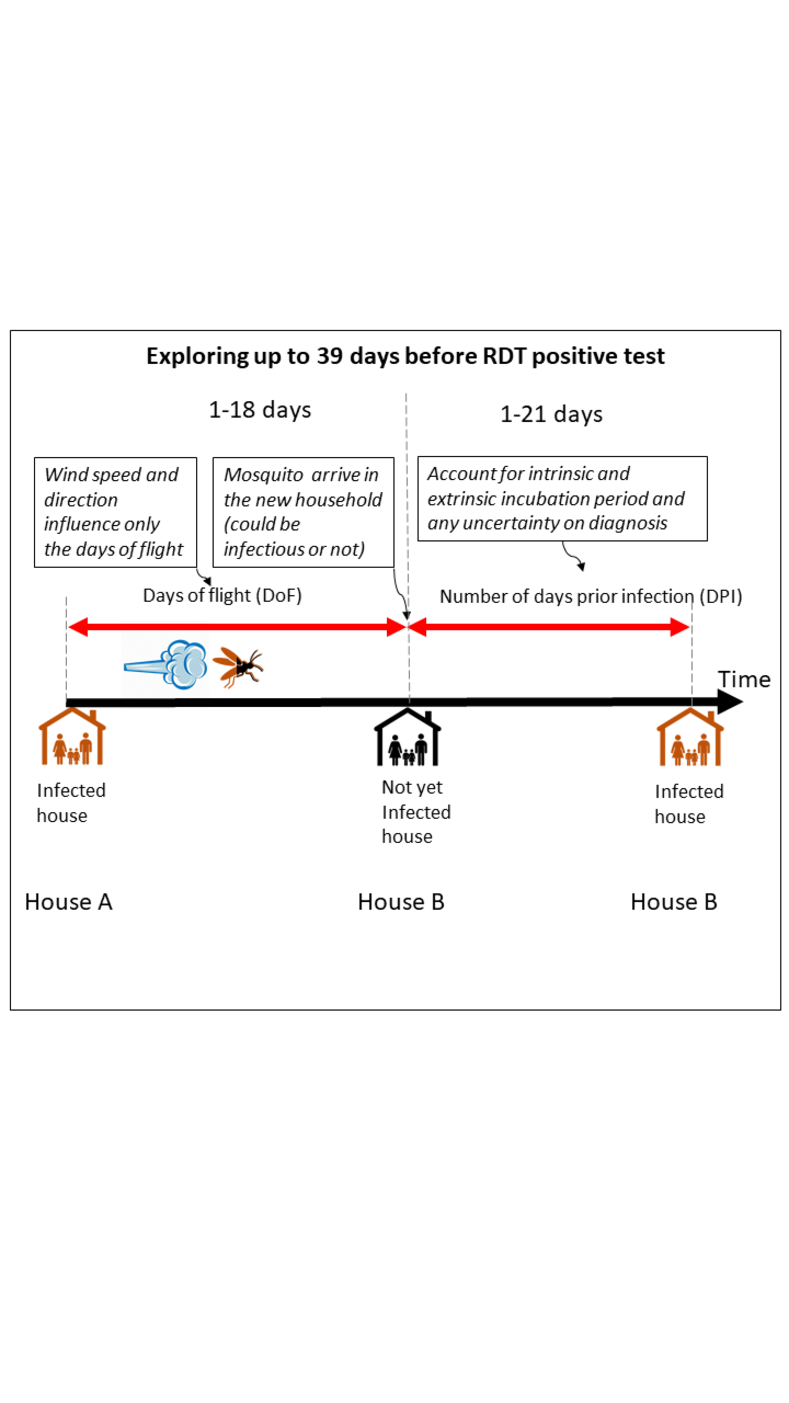

Supplement: S1 Fig — (TIF) [file ppat.1010622.s005.tif]

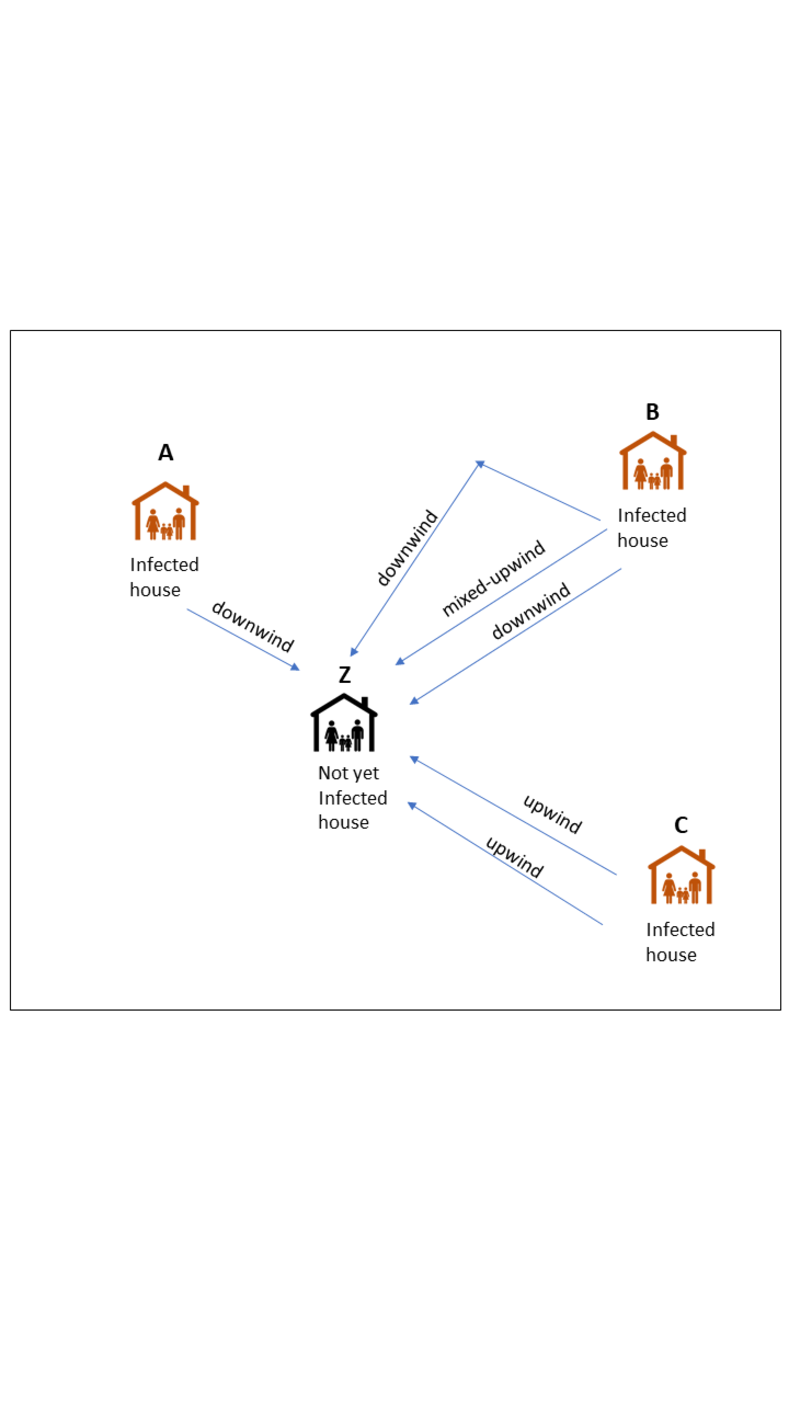

Supplement: S2 Fig — Assuming mosquito survival probabilities and grid probability (of presence of infected houses) are the same for houses A, B and C, and given the fact that the number of simulations are the same for each house, and assuming the potential connections to be 10, the probability that house Z is infected from all surrounding infectious houses is 6/10 (1 connection from A, 3 connections from B and 2 connections from C); the probability that house Z is infected by house B is 3/10; and finally the probability that house Z is infected by downwind movement is 3/10. (TIF) [file ppat.1010622.s006.tif]

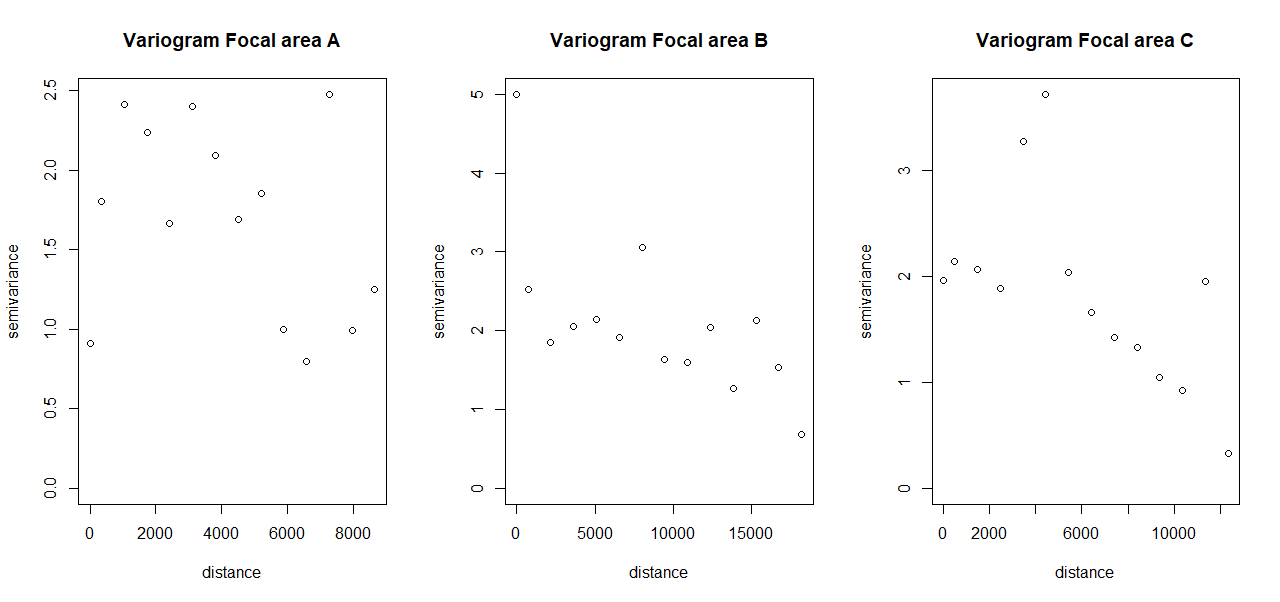

Supplement: S3 Fig — This outcome has been modelled using a Poisson generalised linear mixed model. The Poisson generalised linear mixed model was not corrected for spatial autocorrelation since the experimental variograms show a large nugget effect, i.e. the semivariance does not increase with distance but tends to be stable or decrease (which means that locations further apart looks more similar that locations close to each other). (TIF) [file ppat.1010622.s007.tif]

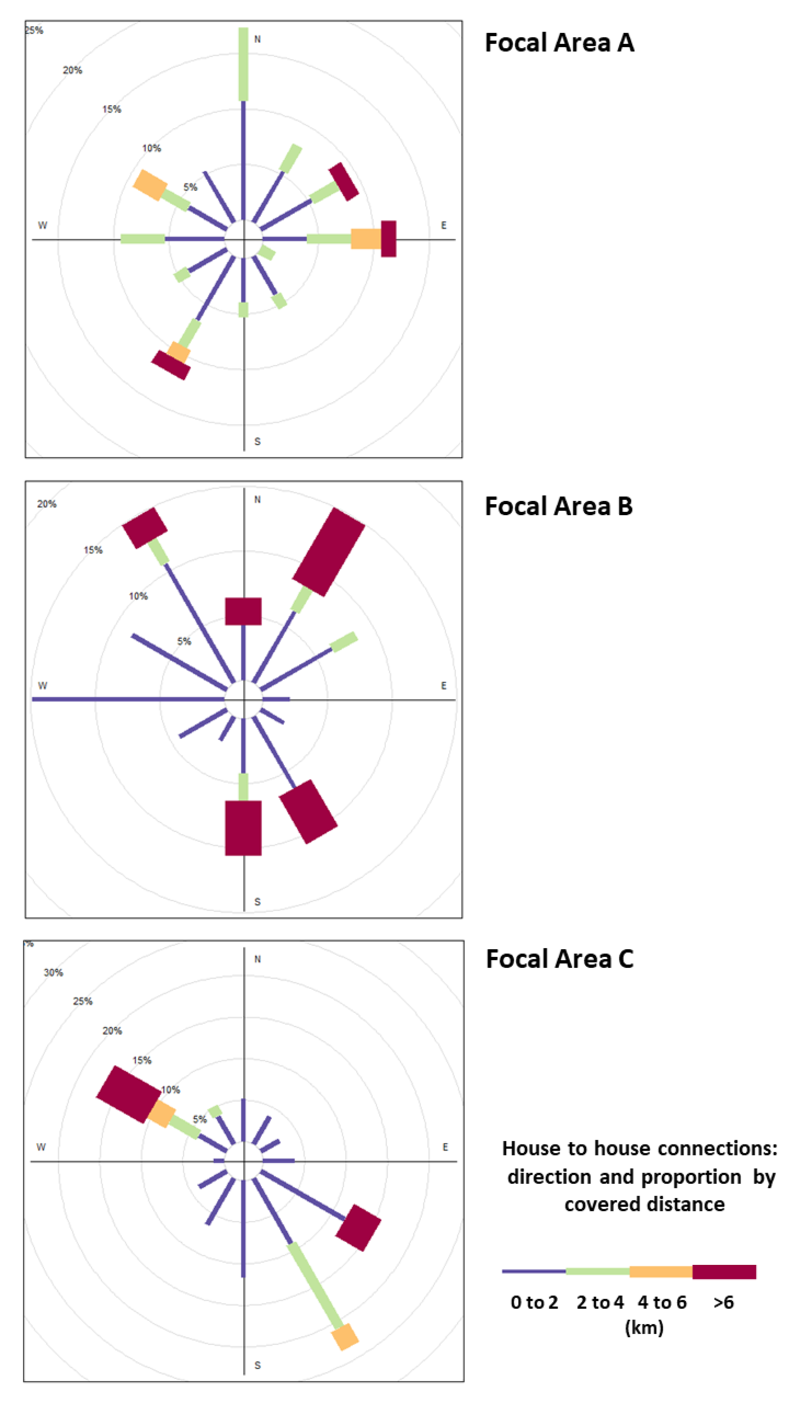

Supplement: S4 Fig — (TIF) [file ppat.1010622.s008.tif]
