# Supplementary material for: Hotspots and super-spreaders: Modelling fine-scale malaria parasite transmission using mosquito flight behaviour
Source: PLoS Pathog. 2022 Jul 6;18(7):e1010622. doi: 10.1371/journal.ppat.1010622 (PMC9292116; doi:10.1371/journal.ppat.1010622)
Supplement: Attachment — Submitted filename: Sedda_PLoS_Pathogens_PointToPointReplies.docx [file ppat.1010622.s012.docx]

# Point to point replies to reviewers

We are grateful to the reviewers for their comments which helped in improving the methodology and the quality of the paper. We also thank the Editors and Reviewing Editors for giving us the opportunity to revise the manuscript.

Below we addressed the reviewers’ comments and suggestions. In **bold** is shown our reply to the comment while in *italic* the text as changed in the manuscript. We have re-ordered comments by Reviewers, while keeping the three reviewing parts clearly labelled to simplify the revision by reviewers.

Line numbers refer to the clean version only.

# Reviewer's 1 Responses to Questions

## Part I – Summary

Reviewer #1: (No Response)

## Part II – Major Issues: Key Experiments Required for Acceptance

[R1] The structure of the article with the Introduction section followed by Results requires some extra explanation for clarity. The authors should briefly describe some of the concepts (connection, hotspots, infected houses…) in the Results section, otherwise it’s difficult to read in such order.

**Thanks, we fully understand this point that has been raised by Reviewer 2 as well. Therefore, and in compliance with the Journal’s formatting guidelines, we have moved the methods section before the results. In addition, we have included additional definitions as algorithm assumptions, survival probability and other probabilities, mosquito movements, etc… .** **Finally, we have added a paragraph explaining in brief the main steps of MALSWOTS calculations:** *In brief, MALSWOTS algorithm has four stages. Firstly, identification of parameter values (such as for DPI, DoF and time of the day considered) that maximise the correlations between mosquito trajectories and house infections trajectories at each house for each plausible temporal scale. Once these parameters are found, the top 5% of the models, ranked by the largest correlation, are averaged (second stage). In the third stage, MALSWOTS simulates the outbreak by re-calculating the mosquito trajectories according to the averaged parameters and a stochastic wind field.* *Each infected house in each interval is associated with many different possible infected mosquito trajectories going from it. Some of these trajectories will end up at uninfected houses and eventually infect them. In the last stage, MALSWOTS examines the associations between suspected movement of infected mosquitoes from infected houses to susceptible houses, and identifies the most likely (highest probability) source and route of infection to each susceptible house, providing the probabilities described above.* (Lines 317-329)

[R1] Although it is undoubtable that mosquito dispersal is an important component of malaria transmission, my main question is if hotspots and super-spreaders predictions would be different in the study area if only epidemiological data were used (i.e., with a simpler model).

**The reviewer raised a very important point. Using only the epidemiological data will mean to remove the daily mosquito survival probabilities from the calculation of the probability that a connection has been made between an infected house and a not-yet infected house by a theoretical mosquito movement/flight. We would like to recall that the entomological data is used only for calculating the survival probabilities by time (not space, since not enough data has been collected) for each area. While we understand the importance on looking at how hotspots and super-spreaders may change with survival probabilities, we wanted to go further than that and check the effect of removing survival probabilities on the model inference and accuracy of predictions, hence if the model does not improve the relative hotspots and super-spreaders locations/numbers are of less interest.**

**The analyses without survival probabilities found a larger proportion of transmission associated to the winds from 6pm and 10pm than in the model with survival probabilities** **(Supplementary Table S3). In terms of validation, the proportion of corrected predictions decreases (from 66% to 55% in Focal area A, and from 55% to 50% in Focal area C), as well as the probability of infection: 0.61 to 0.49 in Focal area A, 0.49 to 0.41 in Focal area C. Focal area B is not affected by removing the survival probabilities (Supplementary Table S4). We have added the following paragraph in the Discussion section:** *We also explored the effect of removing survival probabilities from MALSWOTS. In terms of parameters, the major difference is the shorter DPI for Focal area A (Supplementary Table S2) than in the model with survival probabilities. Validation results show that the proportion of corrected infected houses and the probability of infection are worse in Focal area A and C but unchanged in Focal area B (Supplementary Table S3) when mosquito survival probabilities are not included. This shows that the inclusion of mosquito survival probabilities increases the accuracy of the predictions. However, this seems not the case for Focal area B, probably due to the shortest time of ‘Days to new infection’ among the three areas (Table 2).* (Lines 573-580)

[R1] It seems to me that hotspots are defined around areas with infected houses and super-spreaders are those in which house members experienced more than average number of malaria episodes. Moreover, considering the low number of Anopheles mosquitoes that were captured (1 mosquito/1520 trap collections, 141/1407 and 103/1375 for Areas A, B and C, respectively—Table S1), I wonder if infections are locally or elsewhere acquired, especially in Area A where only one mosquito was collected. Please, clarify it.

**We agree with the reviewer that this point needs a clarification. Elsewhere acquired infection can contribute to the uncertainty in our parameter estimation. However, by exploring thousand potential daily connections between houses (for example for 100 houses the potential number of connections are 100*99/2 = 4950 each day for unconstrained data) over many spatial and temporal scales (450 scenarios) can be assimilated to a probability bias-analysis to control for confounding. Since the optimal parameters are based on maximising the correlations between mosquito flight (constructed from wind data, not entomological data) and infection geographical pattern over hundreds of thousands of connections, possible not-in-house (or its neighbourhood) infection happening in a different location (bearing in mind that we are considering a buffer around the house where infection can still happen) may make the signal (our parameter estimation) weaker (enlarging the confidence intervals) but not biased. The following sentence has been added to the Discussion section in the paragraph regarding the limitations raised from factors not included in this study:** *MALSWOTS analyses thousands of potential connections within different time and spatial scales independently from each other. This balances time-dependent or spatially-dependent uncertainties originating from the factors not considered in this analysis and control for biases and confounding*. (Lines 691-694)

**Added reference: Thommes, E. W., et al. (2020). "Assessing the prior event rate ratio method via probabilistic bias analysis on a Bayesian network." Statistics in Medicine 39(5): 639-659.**

[R1] The authors assume that once one infection was recorded, any subsequent infections occurring one or more months later were independent from the initial one. Hypothetically, a second infection (occurring one or more months later) could be generated by a mosquito that gets infected from either the index case or from another house member who were infected later and not surveyed. In this sense, malaria infections might be persistent over time within a house (being the sink and the source at the same time). It is not clear for me if the algorithm accounts for this hypothetical situation.

**The decision to consider as ‘new re-infection’ only the ones from 30 days from the previous infection, was taken after observing that the best selected models are characterised by shorter than a month infection persistence at house level as expressed in terms of the sum of DPI with DoF ranges.**

**This result is also justified by the negligeable number of short-term reinfections (within 30-60 days from first infection) found in our dataset: only 1 house in each area (see updated Table 1). The rest of re-infection are within 121 to 394 days between infections in area A; 128 to 548 days in area B; and 125 to 363 days in area C. These large time intervals are highly unlikely to come from persistent infection at house level over time, but more likely to have independent source.**

**We have not found any literature on the timescale of recurring malaria (and its potential source) at house level. Therefore, studies are necessary in order to understand the within house malaria dynamics and to implement correct parameterisation in our models.**

**The following changes were made in the manuscript:**

- **We added the information about the ‘number of houses with recurring infection within 30-60 days after previous infection’ in Table 1.**
- **We added the following sentence in the Results section:** *For the latter only 1 house in each area experienced recurring cases within 30 to 60 days after the previous infection, while the rest of the recurring cases were after 121 days from previous infection (Table 1).* (Lines 417-420)
- **We added the following sentence in the Discussion section:** *Missing RDT tests for house members not included in the target population could have caused the underestimation of malaria persistence over time within a house. MALSWOTS explored persistence up to a month after previous infection at each infected house and identified best models with events happening at temporal scales lower than a month (Table 1). It is therefore unlikely that the persistence of malaria for 1 or 2 months in the same house may have played a central role in the malaria spread, while longer recurrences (after 4 months from previous infections) are likely to be independent from the previous infection.* (Lines 670-677)

[R1] Additionally, how was the mosquito survival probability estimated? Please, clarify it.

**The calculation of the survival probabilities is reported in the Supplementary material file (page 7. We briefly recalled the methods in the Methods section (under MALSWOTS heading):** *Mosquito survival probabilities are obtained from a Poisson log-linear model based on exposure time and climate. The survival probability is then measured as expected survival rates per exposure time and climate (full model specification described in page 7 of the Supplementary Materials).* (Lines 302-305)

[R1] The authors say that MALSWOTS correctly predicted that the majority of those houses used for validation (10% of the original data) were infected during the DPI+DoP time. 10% of the original data also contains uninfected houses (and perhaps they are the majority of them). It seems to me that a good prediction power would be the one predicting infected as well as uninfected houses. The authors define “corrected predictions” as the probability of a house which gets infected during a specific period is larger than the probability before or after that the same period. Do the authors also evaluate the alg prediction for houses that didn’t record any infection? Please, clarify it.

**Yes, and thanks for pointing this out, it is true that a large number of false positive may undermine the algorithm. We are now providing the statistics for the uninfected houses in the new table 5:**

*Table 5. Cross validation results for uninfected houses.*

| *Focal area* | *False positive (%) (infected only if connections with probability >0.025)* | *False positive (%) (infected only if connections with probability >0.25)* | *Overall probability to be infected* |
| --- | --- | --- | --- |
| *A* | *37* | *0* | *0.044* |
| *B* | *32* | *0* | *0.084* |
| *C* | *53* | *0* | *0.038* |

**As can be noted from comparing the two tables (Table 4 and Table 5), the statistics used for the uninfected houses are different than those for the infected houses, because uninfected houses are always uninfected. Therefore, we calculated the probability to be infected along the full period (2*(DPI+DoF) prior to 2*(DPI+DoF) after a RDT positive test). We have explained these calculations in the Methods (MALSWOTS validation):** *For the uninfected houses, since these are always uninfected during the study period, we have not calculated probabilities for the period before, around or after a RDT positive test. Instead, we have calculated the probability to be infected during the whole period spanning from 2*(DPI+DoF) days before and after a RDT positive test related to the closest infected house. From this probability we have produced the number of false positive when considering probabilities of connections above 0.025 and 0.25.* (Lines 407-413)

**We described this table in the results:** *False positives for uninfected houses were around a third in focal areas A and B but half for Focal area C. However, these values reduced to 0 when considering a more conservative probability of success of infection (Table 5).* (Lines 520-522)*.*

**We added this sentence to the Discussion section:** *In addition, false positive uninfected houses were absent when using probability of connections above 0.25 (equivalent to 1 successful connection for every 4 potential connections).* (Lines 570-572)

**Finally, we apologise for the typo, but it should be DoF not DoP and we have corrected it throughout the manuscript.**

[R1] Also clarify Table 4 legend—"leaving out 10% of infected houses”—and define objectively what is the meaning of “probability around the time of infection”.

**We have changed the table caption from** ‘Table 4. Cross validation results from leaving out 10% of infected houses and simulating infection connections using the remaining 90% of infected houses.’ **to** ‘Table 4. Cross validation results for infected houses’**. We also added the definition of ‘probability around the time of infection’ in the Table:** *The probability around time of infection is defined as the probability to be infected during one DPI+DoF before and after a RDT positive test.* (Page 16)

[R1] Table 1 presents average and standard deviations of hourly values for some measures. Note that some of them are clearly not normally distributed in such scale and so, mean and standard deviation are not very informative in these cases (e.g., mean and sd equal to 0.09 and 0.99, respectively). It would be more informative to either rescale or to present a different measure of dispersion as, for example, the 95th percentile. Also, it would help readers if the authors presented the percentage for the “number of uninfected houses”, “number of houses infected only once” and “number of reoccurring infected houses”.

**Corrections made.**

[R1] According to the authors, Table 2 shows that wind within 6pm to 10pm time window accounted for between 39% to 57% of the “top 5% of the strongest correlations between mosquito movements and malaria spread from house to house” (lines 148-152). It is not clear for me if the whole Table 2 is based on the “5% of the strongest correlations”. Although this information is found on the supp material where the authors write “top correlations” and on the MALSWOTS alg description (“Take the top n”), it is not clearly specified. Please clarify it on the table legend and also on the methods section.

**Yes, Table 2 is** ‘*obtained from the top 5% of the models ranked by largest correlation*’. We have specified it in Table 2 caption. **We also added a sentence to the Methods (MALSWOTS section):** *For this study we averaged the top 5% of the models ranked by largest correlation.* (Lines 308-309)

[R1] Also regarding Table 2, the proportion of super-spreaders—what it seems to me to be one of the main results of the paper—is informed as a proportion “out of infecting houses”. This result would be better presented as a proportion of all houses, i.e. 10-20% as informed on the text (line 180).

**We have added the information for all the houses (infected + uninfected) to Table 2. Also, we adjusted the figures in the Results section from** ‘is around 10-20%’ **to** ‘*is between 5% and 13%*’. (Line 459)

[R1] Figure 2 seems to not follow the most standard presentation (explanatory variable on the x-axis and response variable on the y-axis). The authors should consider inverting the axis for better interpretation of the figure.

**Correction made.**

[R1] The authors say “The locations of the super-spreaders are shown in Figure 5.” (lines 180-181). I was not able to locate super-spreaders in Figure 5—the figure legend indicates infecting houses.

**We apologise, this was a typo, we were referring to Figure 3 and not Figure 5. We have corrected the text.** (Line 460)

[R1] In the same figure, (i) it is also not stated the meaning of the “unexplained” black circles; (ii) lat and long label axis should be corrected, and; (iii) the number of plotted arrows makes it difficult to obtain any information from the figure. Also, how are downwind and upwind defined?

**Figure 5 wants to show the connectivity structure for each area, and that was our main scope, although we agree this undermine other information that is, however, provided in the other figures and tables. We made the correction for the latitude and longitude and we have added the explanation for the ‘unexplained’ in the figure caption:** *Unexplained (black circle) are houses that are not analysed because outside the spatial and temporal scales considered by the best models.* (Page 19)

**Figure 5 does not show upwind and downwind movements. The flight speed definition of these movements is provided in the supplementary file. We have added the following paragraph in the Methods (Mosquito flight parameters section):** *MALSWOTS considers three mosquito flight movements and their combinations: downwind when the mosquito flight direction is coincident to that of the carrying wind (passive flight); upwind when the mosquito flight against the wind flow and usually in response to host odours (active flight); and random, i.e. irrespective of the current wind speed and direction, or the presence of wind turbulence (active flight).* (Lines 280-284)

[R1] Regarding hotspots (Figure 3), the authors say that “92% of super-spreaders in focal area A were within a 1km radius of an additional super-spreader (a condition we used for hotspot definition).” Figure 3 presents squares and not circles.

**We have removed the reference to radius and corrected the sentence with:** *within a 1km^2^ of an additional super-spreader*. (Line 462)

[R1] How is the position (or center) of the hotspot defined?

**The position of the hotspot is defined manually as containing the maximum number of super-spreaders. We have added this explanation in Figure 3 caption:** *Allocation of the hotspots has been performed manually and capturing the largest number of super-spreaders within each hotspot square.* (Page 17)

[R1] Why do the authors affirm that low transmission areas are relatively small? With the exception of Area C, it seems to me that the majority of houses are located out of the defined hotspots.

**The reviewer is correct; therefore, we changed the sentence from** ‘The low transmission areas are relatively small (Figure 3).’ **to** ‘*The low transmission areas are relatively small in Focal area C (Figure 3)*.’ (Line 465)

[R1] How do the authors measure effectives for the wind-aided/random flights (line 172)?-

**We replaced** ‘was effective’ **with** ‘*accounted for the majority of the connections*’. (Lines 448-449)

[R1] Why could the primary material of the wall structure only be used as a random effect in the model? (lines 193-194)
**We have added the reason at the end of the sentence:** ‘*because not significant as fixed effect*’. (Lines 473-474)

[R1] I was not able to open supp videos 1, 2 and 3.

**The link has been added in the supplementary information. They are available here:** [**https://doi.org/10.17635/lancaster/researchdata/524**](https://doi.org/10.17635/lancaster/researchdata/524)

[R1] Line 212: Should it be Figure 4 instead of Figure 2?

**Sorry for this, it was Table 2 and not Figure 2. Thanks.**

[R1] Line 218: What do the authors mean by “distance between two houses”? Would it be the mean distance between two randomly selected houses? Please, be more specific.

**We changed the sentence to read as:** *In Focal area A, 90% of connections are up to 20 times longer than the corresponding straight distances between the infecting and infected houses.* (Lines 507-508)

[R1] Line 309: Reference out of standard.

**Correction made.**

[R1] Line 523-524: Please, indicate the motivation to use the K-funtion test.

**We have changed the paragraph in order to explain why we use the K-function:** *To estimate potential clustering in house locations (independently from their infection status), complete spatial randomness was tested by employing a K-function test which is independent from the shape of the study region and takes into account the density of the events. The latter allows for comparison among groups regardless the event prevalence.* (Lines 350-354)

**Added reference: Pfeiffer, D. U., et al. (2008). Spatial analysis in epidemiology, OUP Oxford.**

## Part III – Minor Issues: Editorial and Data Presentation Modifications

Reviewer #1: (No Response)

# Reviewer's 2 Responses to Questions

## Part I – Summary

Reviewer #2: This work describes a study that characterizes the role of flying and (super) spreading in malaria infections for mosquitoes. Very few works take approaches with these kinds of parameters. Therefore, such studies have potential for contribution to the field. In this manuscript, the data were rich since datasets were on entomological data, epidemiological and climate (wind, temperature and more), although the number of collected mosquitoes were low - more about that below. Some aspects deserve more explanation. Most important the method for adjusting parameters and finding superspreaders and clusters, due to low numbers of collected mosquitoes and rare infection detection.

**We thank the reviewer for the positive assessment of the novelty of our work. We have amended the manuscript in response to the concerns raised by the reviewer as detailed below.**

## Part II – Major Issues: Key Experiments Required for Acceptance

[R2] As already described, the datasets provide rich information for understanding clusters and the role of flying in dissemination of P. falciparum. However, the low number of collected mosquitoes and limited detection of infection in field mosquitoes is usually the rule in these entomological studies. The algorithms for detection of clusters and quantifying the flight movements are interesting but seem to require a good amount of information to be able to do estimations. The results still require some convincing evidence connecting the number of collections and the inferences. My main concerns are with a need in the Results to provide this link between entomological data and the results drawn from Figure 5. Also, the methodology should provide how the algorithm accounts for this kind of entomological dataset.

**This question is similar to Reviewer’s 1 concern about the importance of entomological data in MALSWOTS, therefore part of this question has been replied in the first question of reviewer 1.** **We modified the Methods (MALSWOTS section) to make clear that mosquito counts are only used for the calculation of mosquito survival probabilities. The main assumption made by MALSWOTS is that each infected house contains mosquitoes that will move to other houses. The amount of mosquitoes is independent from the mosquito catches, and MALSWOTS assumes that at least one mosquito depart from each infected house each day. Not all of them will reach the next infected house, since this will depend on the daily wind patterns. Connection probabilities are therefore calculated on the amount of theoretical mosquitoes reaching the house over the total amount of theoretical mosquitoes leaving the infected house(s) (as in Supplementary Figure S3). The text (MALSWOTS section) has been modified as follows:** *As in SWOTS, MALSWOTS assumes that each infected house contains mosquitoes that are (or will develop to) infected while moving to other houses. The number of mosquitoes moving from house to house is independent from the mosquito catches (otherwise it will not be possible to model areas without or scarce entomological information as Focal Area A). MALSWOTS assumes that at least one mosquito departs from each infected house each day. Therefore, the entomological data is used only for calculating the survival probabilities by time (not space since not enough data has been collected) for each area under study (depending on local environmental characteristics).* *Mosquito survival probabilities are obtained from a Poisson log-linear model based on exposure time and climate. The survival probability is then measured as expected survival rates per exposure time and climate (full model specification described in page 7 of the Supplementary Materials).* (Lines 294-305)

[R2] The maps do not show density of human population. A simple explanation for clustering cases would be spots with human presence, in which a transmission from one house to more than one house should be expected. I recommend more investigation on the role of density of human population.

**Many thanks for raising this important point. We have added the population counts extracted from WorldPop in order to investigate any association with super-spreaders houses. We have added the following sentence to the Methods (Investigating risk factors of super-spreaders houses section):** *Finally, the unconstrained population counts (because of the low accuracy of building satellite information for the Majete rural area) for the year 2016 at a resolution of 3arc (approximately 100m at the equator) from WorldPop, has been included as covariate.* (Lines 385-387)

**Added reference: Tatem, A. J. (2017). "Comment: WorldPop, open data for spatial demography." Scientific Data 4**

**We have described the result of including the population counts in the Results section:** *Population counts were not significantly associated to super-spreaders (OR 1.06, 95% confidence interval: 0.98 and 1.15), and the model explained 55% of the total variance when this predictor was included (therefore this predictor was removed from the model described in Table 3).* (Lines 474-477)

**Finally, we have added the following sentence to the Discussion section:** *By contrast, population counts were not significantly associated to super-spreaders, although as shown in different studies this is linked to malaria risk.* (Lines 596-598)

**Added reference: Kabaria, C. W., et al. (2017). "The impact of urbanization and population density on childhood Plasmodium falciparum parasite prevalence rates in Africa." Malaria Journal 16.**

[R2] The manuscript is written with methodology in the end. This made it more difficult to understand. Efforts are necessary to make a better flow and understanding of the results.

**Since this was also pointed out by Reviewer 1, we have decided in compliance with the Journal’s formatting guidelines, to move the methods section before the results and increase explanations in the methodology and results sections (see our reply to Reviewer 1).**

[R2] The term "super-spreader" tends to be overused and might have different interpretations. In this manuscript, the Introduction describes the super-spreader as accounting for a high proportion of the transmission. My perception from the analysis in Table 2 is that super-spreader means infecting more than one house. The, in the Results section, the definition becomes when infecting two or more houses. This should be stated clearly in the Introduction and be consistent throughout the manuscript.

**We agree with the reviewer and we hope that with the methods before the results will remove any confusion about our definition of super-spreaders. In the Methods section we have changed the hotspot definition section in ‘***Hotspot and super-spreaders definitions***’. In this section we added:** *Our definition of a super-spreader is ‘a house that is the source of infection for two or more other houses within the study period (07/2016 to 05/2018).* (Lines 360-361)

**In addition, we have added the definition of super-spreaders in the Introduction:** *In this study a super-spreader is a house infecting two or more other houses.* (Lines 89-90)

[R2] In the Results section, what is the probability in the less conservative scenario as opposed to the probability of 0.25? The assumed probability in the previous scenario should be stated.

**It is the probability above 0, in which case even a single mosquito trajectory reaching an uninfected house could be the cause of malaria transmission. We have re-worded the previous sentence in:** *Wind-aided mosquito movements explained 91%, 85% and 71% of the possible house-to-house malaria transmissions in Focal area A, B and C respectively, with a probability above 0* *(for example if this probability was 0.01, out of 100 infected mosquito wind-based trajectories departing from an infected house, at least 1 would reach an uninfected house and would subsequently become infected).* (Lines 435-6439)

[R2] In Table 3, what means "Treatment - out"?

**We have added a note in the table to explain treatment-out:** *If yes, the village has the same interventions as the control village but it is excluded from the trial analysis because of proximity to other villages.* (Page 15) **In addition, we have provided more information about control village in the Study area and period section:** *The MMP included a cluster-randomized controlled trial from May 2016 to May 2018. Of the 65 villages, 12 were excluded from the treatment arm allocation to reduce the risk of contamination between different treatment arms. The remaining villages were assigned to one of four groups: a control arm; house improvement (HI); larval source management (LSM); and HI+LSM. NMCP interventions and community engagement were used in all arms.* (Lines 183-186)

**Finally, to simplify the jargon, we have replaced the term ‘out’ with ‘excluded’.**

[R2] I suggest changing "Goats present" to "Presence of Goats (YES/NO)". If it is number of goats, please define it.

**We have made the change as suggested by the reviewer (Presence of Goats (YES/NO).**

[R2] Many authors use DPI as "days post-infection". In this manuscript, however, DPI means "days of previous infection". What is the definition of DPI? Days between adult emergence and the event of infection?

**We apologise, this was not defined in the main manuscript but only in the supplementary information. We have updated the Methods in the ‘Mosquito flight parameters’ section by identifying the acronyms for each parameter and adding more information on the DPI:** *DPI accounts for the full intrinsic incubation period, uncompleted extrinsic incubation period (if not completed during the days of flight) and uncertainty in RDT test.* (Lines 276-278)

[R2] What is DoP? I see in the Table, DoF, Days of mosquito Flight.

**Yes, this was a typo sorry. It should be DoF not DoP and we have corrected it throughout the manuscript.**

[R2] Figure S1 gives a notion of these time intervals. Still, some values seem high. For instance, 29 days to new infection would mean a mean generation time of 29 days? It seems a long interval and should be discussed.

**Indeed it is possible that houses connections range from 1 to 39 days. However, this value must be decomposed by two terms: days of mosquito flight (how many days the mosquito takes to reach the next house) and number of days prior infection which takes into account the time from mosquito arrival to bite, including completion of extrinsic incubation period, and time for the development of malaria in the person. MALSWOTS explore all possible combinations of the range of these two parameters in order to optimise them. 29 days is the value to new infection obtained by MALSWOTS in Focal Area A, however, most of this time is spent in flight (Table 2, 16 days with 95% CI 4 to 16 days). We have added the following sentence in the Discussion section:** Finally, the average time necessary to establish a successful connection between two houses (17 to 29 days) is sufficient for the completion of the extrinsic (in the mosquito) and intrinsic incubation period (in the human host) ^49^. *A great proportion of this time is composed by the mosquito movements.* (Lines 646-649)

[R2] In the methodology, why these number of 72% of houses were randomly selected for mosquito sampling?

**We have added the following sentence to the methods (Entomological data section) to clarify this point:** *Maximum operationally feasible sample sizes for mosquito sampling (n=65 households per focal area) and epidemiological data collection (n=90 households per focal area) were determined during baseline rounds in 2015.* (Lines 245-247)

[R2] I understand that there was low number of collected mosquitoes, but survival probability were based on overall numbers of male and female, and there is a difference in the literature between survival between male and female mosquitoes. How were survival probabilities estimated? Please review the survival probabilities and how it impacts the results.

**This is an excellent point from the reviewer. As per reply to Reviewer 1 we have added more information on how survival probabilities were calculated (see above). The number of male mosquitoes was negligible, only 1 in Focal area B and only 2 in Focal area C (Supplementary Table S1). Therefore, we have removed the male catches from the survival analysis and recalculated the survival probabilities. As expected, these have not changed. We have corrected the sentence in the Methods (Entomological data section):** *The survival probabilities were based on the number of female Anopheles per trap per day*. (Lines 264-265)

[R2] What is "set between day 21 and day 1 before the house survey"?

**That was the DPI. Our algorithm assumes that an engorged (with infected blood) mosquito departed from an infected house between 21 to 1 day before the RDT positive test. We have corrected the sentence in the Methods (Mosquito flight parameters section):** *number of days prior to a new human infection (DPI) (r), that has been set between day 21 and day 1 before the date of the house survey.* (Lines 270-271)

[R2] Also, I expect some dependence on temperature for some parameters, such as the extrinsic incubation period. This should be discussed.

**We have extended the limitations section in the Discussion section by adding:** *In addition, climate (e.g. daily temperature fluctuation) does not only influence mosquito survival but also parasite growth and development and mosquito behaviour, hence further development of MALSWOTS may include additional probabilistic components (in addition to the existing mosquito survival probability and mosquito movement probability) related to these factors.* (Lines 681-685)

**Added refences:**

**Suh, E., et al. (2020). "The influence of feeding behaviour and temperature on the capacity of mosquitoes to transmit malaria." Nat Ecol Evol 4(7): 940-951.**

**Paaijmans, K. P., et al. (2010). "Influence of climate on malaria transmission depends on daily temperature variation." Proc Natl Acad Sci U S A 107(34): 15135-15139.**

## Part III – Minor Issues: Editorial and Data Presentation Modifications

[R2] Abstract. The abstract is purely descriptive, without providing any of the assessments, which I think could be provided.

**Abstract has been re-worked providing details on the main findings.**

[R2] Table 1. Character "^" signals confidence intervals but there is only one number in parentheses.

**Sorry, we have reported the numbers without space, we have now added a space between the two numbers in both Table 1 and Table 2.**

[R2] Figure 5. What is calm (bottom right)?

**We have added the explanation in the figure caption:** *Calm refers to winds below 0.5 m/s.*

# Reviewer's 3 Responses to Questions

## Part I – Summary

Reviewer #3: This paper presents an approach to determine the location of malaria hotspots and super spreader houses, using statistical modelling of infectious mosquito movements from house to house, integrating infection and wind data collected as part of a larger malaria study in the region of southern Malawi. Their model is able to determine key components of transmission of malaria in a rural setting, including stable and unstable hotspots of transmission, and identify key parameters such as the speed of spread between households, the role of wind factors, and mosquito movement parameters. It seems with this quantification control design for malaria in such a rural setting can be enhanced and optimized. I found the paper in general well-written, and clear, with interesting results presented in a coherent manner and supported by their analyses. However immunological factors which certainly play a role are not included and the differences and similarities between the different areas A, B and C could have been discussed more. Similarly the predictive ability of this modeling framework can be elaborated upon. In general I have only a few clarification points.

**We thank the reviewer for the positive assessment of the novelty of our work. We have amended the manuscript in response to the clarification points raised by the reviewer as detailed below.**

## Part II – Major Issues: Key Experiments Required for Acceptance

Reviewer #3: (No Response)

## Part III – Minor Issues: Editorial and Data Presentation Modifications

[R3] 1. In my view the introduction deserves a bit more background on the epidemiology of malaria in the region that is studied, in southern Malawi or Malawi in general. What are the trends, what are the control options that are being implemented, what are the numbers of malaria. This context is needed. It is partly present in the Methods, but needs to be in the Introduction as well.

**We have added the following sentences to the Introduction section:** *Malaria endemicity in the study area is high but heterogeneous with identifiable hotspots of transmission [27]. Transmission of the malaria parasite Plasmodium falciparum occurs year-round, with annual peaks typically following the rainy season [28]. From 2015 to 2018 the prevalence of P. falciparum infection in children aged 6-59 mo. during repeated cross-sectional surveys varied within and between years, from a low of 5% in December 2016 to a high of 60% in June 2015. The Chikwawa District Health Office implements malaria interventions in the district for the National Malaria Control Programme (NMCP). This includes provision of ITNs to pregnant women and children under 5 years old, mass distribution campaigns of ITNs, intermittent preventative therapy for pregnant women, and malaria case diagnosis and treatment with artemisinin-based combination therapy. The only mass distribution of ITNs during the study period was in April 2016. PermaNet 2.0 (Vestergaard Frandsen, Lausanne, Switzerland), Olyset Net (Sumitomo Chemical Company, Tokyo, Japan), and Royal Sentry (Disease Control Technologies, USA) were distributed in the district.* (Lines 139-152)

[R3] 2. Table 1 needs to have units for the numbers of infections reported. If the study was conduced over two years, than these numbers need to be interpreted within this time-frame. In a first reading, I found it confusing, and hard to interpret, whether this referred to a snapshot in time or an average over a longer period.

**We have specified the timeframe in Table 1 caption:** *Table 1. Environmental and epidemiological characteristics of focal areas for the period under study (1^st^ July 2016 to 12^th^ May 2018).* **We also specified the units for the number of infections:** *Malaria RDT positive per people surveyed (%).* (Page 6)

[R3] 3. I am not sure the hotspot definition is very clear in the paper. Does it have to do with deviation from the average? Mathematically, what is a hotspot? Please define it clearly.

**There is no mathematical definition of our hotspots and it is simplistically based on the number of super-spreaders within 1km2. To make this clear we have modified the first sentence in the Methods (‘Hotspots and super-spreaders definition’ section):** *Hotspots are usually defined statistically ^35,59^ although no strict definition exists. Here we simply considered a hotspot an arbitrary area of 1 km^2^ (as the geographic size for hotspots described in ^34^) containing at least two super-spreaders.* (Lines 361-364)

[R3] 4. The definition of super-spreaders is presented in lines 177-178, an infected house causing 2 or more than 2 other infections, but what is the average? I see the distributions in Figure 2 (which are a model-derived quantity, right?) but what is the mean? Please also always put the unit of time in these numbers. What is the spatial and temporal denominator of these infections?

**Thanks for the suggestions. We have added this information (and relative confidence intervals) in Table 2 as:** ‘*Average number of infected houses by super-spreaders*’; **and** ‘*Average number of infected houses out of all houses*’. **Table 2 and Figure 2 also include the unit of time, in Figure 2 we have specified that this are obtained from MALSWOTS:**

*Table 2. MALSWOTS estimated optimal parameters and transmission summaries* *obtained from the top 5% of the models ranked by largest correlation. ^ confidence intervals; * see Figure 4 for explanation. All parameters are calculated over the whole study period.* (Page 7)

*Figure 2. Spreaders for each focal area obtained by MALSWOTS for the whole study period.* (Page 15)

**For what concern the spatial and temporal denominator of these infections, the values are averaged over the explored parameters’ ranges. We have added in Table 2 the spatial and temporal scales of the infections originated by the super-spreaders as:** ‘*Median connection distance from super-spreaders to houses (km)*’ **and** ‘*Median temporal distance from super-spreaders to houses (days)*’. (Page 7)

**Also we have included all these findings in the Results:** *On average each super-spreader infected 3 other houses (Table 2) and in comparison to non-super-spreaders they tend to have connections which are longer in time but not in distance (Figure 4).* (Lines 457-459)

[R3] 5. In the part about the investigation of risk factors for super-spreaders, what is the assumption about the space? Is this a non-spatial Poisson process or a spatial one?

**It is a non-spatial Poisson process since the variograms (and overall variogam) showed a large nugget effect. We have included this information in the Methods (Investigating risk factors of super-spreader houses section):** *This model was not corrected for spatial autocorrelation because the outcome number of infected houses originating from each house was affected by a strong nugget effect (weak functional structure in the semivariance, see Supplementary Figure S4).* (Lines 369-372)

**Added reference: Oliver, M. A. and R. Webster (2014). "A tutorial guide to geostatistics: Computing and modelling variograms and kriging." Catena 113: 56-69**

[R3] 6. Are the super-spreaders intrinsically super-spreaders or they are a consequence, a side-effect perhaps of best environmental conditions meeting there for optimal transmission? It seems the paper is suggesting the second interpretation, which in my view violates somehow the definition of a super-spreader, it is more of a “passive” super-spreading than of “active super-spreading”. I think this notion is more accurately described by the concept of hostpots - localized- than “super-spreading” ability which somehow should be independent of space. Please comment or clarify.

**In the Introduction section we have included the definitions and more information regarding the difference between super-spreaders and hotspots. We have added:** *In this study a super-spreader is a house infecting two or more other houses. This means that super-spreading, as defined in our study is dependent of location and house characteristics (and the characteristics of people living in the house). It is dependent on location because the spreading is influenced by the environment where mosquito moves. When an area contains more than one super-spreader for a certain length of time then the area is defined as hotspot.* (Lines 89-94)

[R3] 7. In Figure 5, can the left panels be represented in terms of their angular distribution and matched with the wind direction frequency distribution on the right for comparison?

**We have kept Figure 5 unchanged, but we have added a supplementary figure (S5) with the angular distribution of the connections. The following sentence has been added to the Results section:** *There is an agreement between the directions of the connections (Supplementary Figure S5) and the wind directions (Figure 5 left panels) in Focal area B and C, less for Focal area A where the upwind and downwind mixed movements have a strong random component (Table 2).* (Lines 504-507)

[R3] 8. Immunological aspects are not touched upon at all in the study. Are the identified superspreaders always super-spreaders over time? Don't these families acquire immunity to malaria and then stop being super-spreaders at some point? Any comments on this phenomenon, perhaps happening over longer timescales than the ones modelled in the study would be relevant, and strengthen the paper. How could immunological factors of the human population be integrated?

**Yes, MALSWOTS does not account for immunological aspects. We use real data and infected houses are those RDT positive meaning that our epidemiological unit is indeed infectious for a certain time after RDT positive test.**

**Are the identified superspreaders always super-spreaders over time? No, super-spreaders are super-spreaders only within a pre-defined period coincident with DPI days before RDT positive test to DoF days after positive RDT test. For example for area C (Table 2) and for the average values of DPI and DoF, if an house had an RDT positive test the 20th of September, a mosquito bite is likely to have happened from the 14th of September (DPI = 6 days) and transmission to other houses occurring in the next 15 days (DoF = 15 days).**

**Don't these families acquire immunity to malaria and then stop being super-spreaders at some point? Yes, presumably and although this was outside the scope of our study, this may be apparent in future hotspot dynamics (over a period of 5-10 years) if we see stable hotspots shifting to unstable or unclustered areas.**

**How could immunological factors of the human population be integrated? It would be very difficult to integrate immunological factors given the complex dynamics of malaria immunity and large resources needed to screen the population.**

**To summarise, we have added the following sentence in the Discussion section:**  Finally, we have not investigated potential other causes of the strong spatial epidemiological and entomological heterogeneity in the three focal areas which may depend on demographics, housing characteristics, immunity, malaria interventions, mosquito behaviour (related to ecological conditions [23]) and human movements [75-77]. *In particular by not considering immunological factors it is not possible to predict the stability of hotspots over time, since immunity may cause a stable hotspot to become unstable or to terminate.* (Lines 685-690)

[R3] 9. How could this model be used for epidemiological forecasting? If we know the number and distribution of infected houses, mosquito distribution, wind directions and other variables extracted as significant here, can we apply this model to predict disease risk over time across space? I found this aspect a bit under-explored in the paper, for example connecting the differences and similarities between areas A B and C. Can the parameters estimated and reported here be (universally) used for other regions and rural settings, if we know their local variables?

**Thanks for this very important point. We have added the following paragraph in the Discussion section:** *MALSWOTS is a parsimonious and efficient model to forecast local transmission by mosquito movement when forecasted environmental (wind and climate) data is available. In this scenario we could simulate transmission patterns assuming infections starting from a specific house, or if known which houses are infectious MALSWOTS can identify the most likely infected houses by mosquito movements. In the same way, the relative risk (as shown in Figure 6) for the entire area can be forecasted. However, the parameterisation for one area cannot be used for other areas given the intrinsic differences in urban and environmental compositions and structures in each area as shown in this study for the three focal areas analysed. Therefore, each area will require a separate parameters’ inference (as described in stages 1 and 2 in Supplementary Figure S2) before running the simulations (stage 3 and 4 of the same supplementary figure).* (Lines 707-717)
